# Trees in the Eyes of Young Learners: A Study on Knowledge and Educational Methods

**DOI:** 10.1002/pei3.70090

**Published:** 2025-10-29

**Authors:** Daša Bombjaková, Veronika Rusňáková

**Affiliations:** ^1^ Faculty of Social and Economic Sciences, Institute of Social Anthropology Comenius University in Bratislava Bratislava Slovakia

**Keywords:** botanical literacy, ethnographic methods, experiential learning, informal education, peer learning, plant awareness disparity, rural childhood, salience of tree species

## Abstract

Children often exhibit limited knowledge of plant life, a phenomenon referred to as “plant awareness disparity,” which can hinder the development of environmental literacy and ecological stewardship. Despite the foundational role of plants in ecosystems, educational systems and cultural narratives frequently prioritize animals, leaving children's understanding of trees underdeveloped. This study aimed to examine how primary school children in the rural Slovak village of Granč‐Petrovce acquire knowledge about trees, and how formal education, family practices, and peer interactions shape this learning. Using an interdisciplinary approach grounded in ethnobiology and social anthropology, the research combined participant observation, free listing, map drawing, semi‐structured interviews, diaries, and outdoor walks with 10 children aged 7–9 over a five‐week period. Results revealed that children were most familiar with fruit trees such as apple and cherry, with knowledge strongly tied to personal, hands‐on experiences in gardens and during family activities. While formal education introduced certain species, these were less salient unless reinforced through lived interaction. Peer learning also played a notable role, with older children often teaching younger peers about plants. Despite spending time outdoors, children who lacked active engagement with plants showed limited ecological understanding, particularly in areas like tree reproduction. The study underscores that the quality of engagement with nature—especially when culturally and relationally meaningful—matters more than the quantity of exposure. These findings advocate for educational strategies that integrate outdoor, experiential, and peer‐based learning to foster deeper plant knowledge and reduce plant awareness disparity among young learners.

## 1 Introduction

Globally, plants, particularly trees, are often overlooked despite their critical role in supporting life on Earth. This is particularly evident in young children, who commonly struggle to comprehend the complexity of the plant world. The immobility of plants and their perceived monotony, compared to the dynamic nature of animals, contributes to this challenge (Jaun‐Holderegger et al. 2022; Wandersee and Schussler 1999; Tunnicliffe 2001). This phenomenon, known as “plant blindness” (Wandersee and Schussler 1999), has been further conceptualized as “plant awareness disparity” (Parsley 2020), highlighting a widespread lack of recognition of plants' ecological importance. This disparity often stems from the difficulty children face in distinguishing and identifying plants, compounded by a cultural and educational emphasis on animals rather than flora (Jaun‐Holderegger et al. 2022; Stagg and Dillon 2022).

The lack of interest in plants can contribute to a lack of concern for ecological issues like deforestation, further perpetuating ignorance about plant species and their need for protection (Stroud et al. 2022; Wandersee and Schussler 1999). Such a gap in knowledge impedes the development of a positive relationship with nature, which is essential for fostering environmentally protective behaviors. Fieldwork has shown that hands‐on, experiential learning plays a significant role in cultivating positive attitudes toward plants, especially in young children (Zoldosova and Prokop 2006). The more children engage with plants, the greater their ability to appreciate their ecological roles, which in turn increases the likelihood of developing pro‐environmental attitudes in the future (Pyrovetsi and Daoutopoulos 1999).

Despite the recognized importance of plant education, research on children's understanding of plants, particularly trees, remains sparse. McNair and Stein (2001) observed that children often associate the term “plant” with blooming flowers, while they struggle with the broader concept of plant identification and terminology. This is reflective of a larger trend, where children exhibit greater familiarity with animals due to their dynamic and engaging characteristics, as compared to the more stationary and uniform nature of plants (Jaun‐Holderegger et al. 2022; Wandersee and Schussler 1999; Tunnicliffe 2001; Pany et al. 2022). Such preferences may be further influenced by a lack of direct interaction with plants in early life, which has been linked to the development of plant awareness disparity (Balding and Williams 2016). This lack of early interaction may hinder not only knowledge of plant species but also the appreciation of their significance in natural ecosystems.

Research also highlights that early exposure to nature and foundational biological knowledge significantly influence children's environmental attitudes and behaviors in adulthood (Tanner 1980). The transmission of biological knowledge often occurs informally through family experiences (Jaun‐Holderegger et al. 2022), but a lack of engagement with plants in early years can have negative repercussions on both academic performance and the perceived importance of nature (Balding and Williams 2016). Thus, understanding children's knowledge of local tree species and how these species become memorable or interesting is critical in mitigating plant awareness disparity.

Several studies have examined the concept of plant awareness disparity from different perspectives. Ethnobiological studies often employ methods such as free listing to explore the breadth of knowledge that children possess regarding plant species (e.g., Hough and Ferraris 2010). In contrast, biology education research tends to focus on formalized teaching of plant identification and classification (Tunnicliffe 2001; McNair and Stein 2001). Furthermore, social anthropology, through participant observation (Spradley 1980; Emerson et al. 2011), can provide insights into how cultural and familial practices shape children's relationships with plants and nature. This study integrates these diverse research methodologies—free listing, participant observation, and questionnaires—to explore children's tree knowledge and the influences shaping it. By synthesizing these approaches, the study provides a more comprehensive understanding of how children perceive and interact with trees, with particular attention to the cultural and educational influences that contribute to their knowledge of tree species.

This study seeks to expand upon current literature by examining how children's knowledge of trees is not only shaped by formal education but also by informal, experiential learning that occurs within the family and community. Additionally, the study explores the importance of peer learning in the transmission of tree knowledge, highlighting that children often place greater value on the opinions of their peers than on those of adults (Hirschfeld 2002). By examining both formal and informal learning environments, this research aims to contribute valuable insights into the ways children's understanding of trees can be fostered to encourage greater plant awareness and environmental stewardship.

This study also contributes to broader theoretical frameworks in environmental and educational research. First, it aligns with the concept of *ecological literacy*, which emphasizes the importance of developing awareness, understanding, and care for the natural systems that sustain life (Orr 1992). Second, it reflects principles of *place‐based education*, which advocate for grounding learning in local ecologies and cultural contexts (Sobel 2004). By focusing on a specific rural setting and integrating formal, informal, and peer learning, this study bridges these frameworks to explore how children's relationships with trees develop across multiple educational landscapes.

### 1.1 Research Questions

- How do primary school children acquire knowledge about trees, and what are the sources of this information (e.g., formal education, family, community, peer interactions)?
- What is the extent of primary school children's knowledge regarding local tree species, and what factors make certain species more memorable or significant to them?
- How does children's engagement with outdoor activities, particularly those that involve active interaction with plants (e.g., gardening, nature walks), influence their understanding of trees and plant species?

## 2 Materials and Methods

### 2.1 Research Context

#### 2.1.1 Respondents and Locality

**FIGURE 1:**
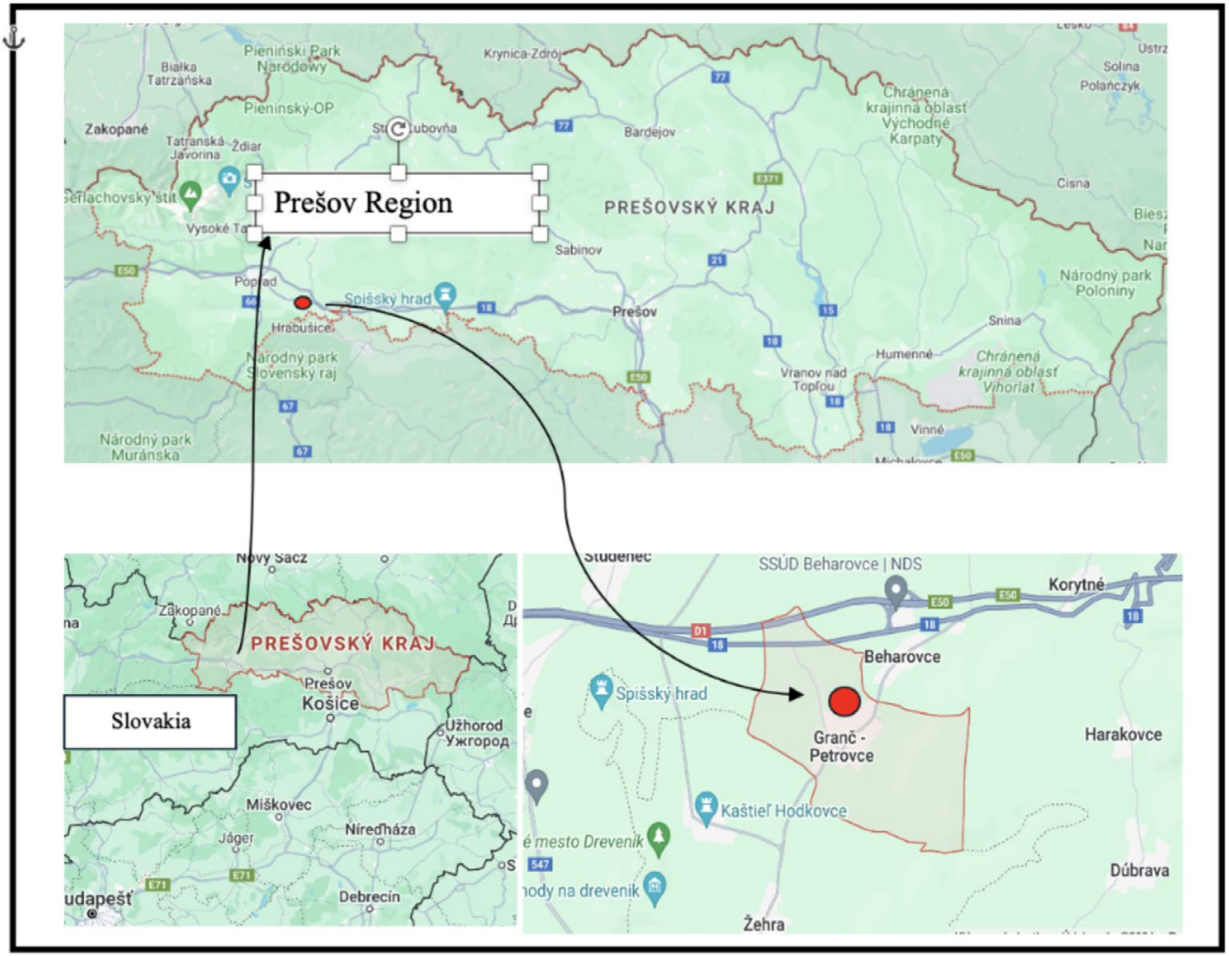
Location of the Granč‐Petrovce, where we conducted the study.

The study was conducted at an elementary school in Granč‐Petrovce, a village in the Levoča district of the Prešov region in Slovakia (Figure 1). The village covers 3.17 km^2^ and has a population of 638 (2020), located at an altitude of 450 m. This rural area is close to nature, where children often engage in farming activities, tending to small vegetable gardens and orchards. The aim of the research was to explore whether children maintain an active relationship with nature, particularly trees. The study took place over 5 weeks, with participants including children from a combined second and fourth‐grade class, their teachers, and parents. Children's ages ranged from 7 to 9.

Granč‐Petrovce was selected as the research site based on both practical and conceptual considerations. We were searching for a rural locality where the majority of inhabitants maintain gardens and orchards, as our research aimed to explore whether direct contact with nature influences children's knowledge of trees. To address this question effectively, it was crucial to focus on a rural rather than an urban area. Eastern Slovakia, in comparison with the more urbanized western part of the country, offers a higher prevalence of rural communities with closer ties to traditional agricultural practices. Granč‐Petrovce, situated in the Levoča district, provided the ideal context: a small village with accessible natural environments, active gardening practices, and a school community open to collaboration. These factors enabled us to investigate how informal, everyday interactions with nature intersect with formal education in shaping plant knowledge among young learners.

The school, located in a 19th‐century manor, is surrounded by diverse natural settings, including mixed forests with various tree species and abundant fruit trees. The school has 31 students, and children are taught in a combined class. Without a gymnasium, outdoor exercise is necessary. Adjacent playgrounds, a sports field, and an outdoor gym provide continued access to outdoor activities after class. The village also has a football field where children play and participate in community events. Observations were conducted in these areas.

The study focused on 10 students: four fourth‐graders (three girls and one boy) and six second‐graders (four girls and two boys). Two teachers, including the school principal, were involved. Informal interviews were conducted with some parents. In terms of sample selection criteria, the class was treated as the primary unit of analysis. All students from the combined second and fourth‐grade class who received parental consent were invited to participate, regardless of gender. Gender balance was not a determining factor. The key criterion for class selection was that the students were already learning about tree species and ecology as part of their school curriculum.

Most children live in homes with functional backyards where families grow vegetables and fruit trees, such as apples, pears, and plums. While gardening is recreational, many grandparents also keep domestic animals and maintain gardens. These families are not dependent on farming for their livelihood but cultivate crops for personal consumption. Some children also have animals like chickens, rabbits, and pigs (Figure 1).

#### 2.1.2 Trees in School Curriculum

Preschool children in the Slovak Republic learn about the role of trees in ecosystems but are not explicitly taught to recognize specific tree species or their practical uses. The focus is on tree ecology and environmental adaptations. By the end of 2nd grade, students should identify various environments and the kind of plants that live within them, understand plant diversity and adaptations, and recognize the importance of seeds, seed dispersal, and plant dependence on the non‐living environment. They should also explain seed dispersal mechanisms and the sprouting process. By the end of 4th grade, students are expected to describe the forest as a community of interdependent plants and animals, identify trees like the English oak, European beech, European hornbeam, and Scots pine, and explain the conditions plants need to thrive. Additionally, they should understand how aquatic plants adapt to water environments and recognize water as a habitat for various animals (Štátny pedagogický ústav 2014a, 2014b).

### 2.2 Research Methods

This study utilizes an interdisciplinary methodological approach, combining ethnobiology, education, and social anthropology to provide a holistic view of children's relationship with trees. These methods allow for a comprehensive exploration of plant awareness disparity among young learners, emphasizing not only cognitive knowledge but also emotional and social dimensions of plant education. The ethnobiological approach, in particular, offers a culturally grounded perspective on plant knowledge that is often absent in traditional biology education. Primary research methods were ethnographic methods—participant observation and interviews with children.

#### 2.2.1 Ethical Procedures

This study was conducted in accordance with ethical standards for research involving children. Ethical approval was granted by the Research Approval Committee of the Institute of Social Anthropology, Faculty of Social and Economic Sciences, Comenius University in Bratislava, in May 2023. Prior to the start of data collection, informed consent was obtained from the parents or legal guardians of all participating children. The school director acted as a key gatekeeper, with all initial communication and meetings facilitated through her. She played an instrumental role in introducing the researcher (VR) to the local community and establishing trust with the school staff and families. Her support was essential in securing parental consent and ensuring smooth collaboration with the school throughout the research process.

In addition to written parental consent, verbal assent was obtained from each child participant. The purpose and procedures of the study were explained to the children in age‐appropriate language, emphasizing the voluntary nature of their participation and their right to withdraw at any time without any negative consequences. To protect participants' privacy, all data were anonymized, and pseudonyms were used in all transcripts and reporting. No audio recordings were made of interviews with children to ensure a relaxed and respectful research environment.

Throughout the research period, informed consent was obtained from the parents of all students in the class, with no withdrawals of consent or assent.

#### 2.2.2 Participant Observation

Participant observation is a crucial research method in social anthropology (DeWalt and DeWalt 2010), enabling researchers to gain deep insights into the everyday lives and practices of the community under study. By actively engaging in the environment, the researcher can observe not only the overt behaviors of participants but also the subtler, unspoken norms, values, and social dynamics that may not be readily apparent through interviews or surveys alone. This method helps establish rapport and build trust (Spradley 1980). Furthermore, participant observation allows researchers to immerse themselves in the context, providing a holistic view of social interactions and enabling them to interpret findings from an insider's perspective. In this study, participant observation was a key method—focused on the primary school. The goal was not just to observe in the classroom but for VR to actively assist the teacher, participate in children's play, and engage in various school activities. Participant observation was essential for understanding the school rhythm and the relationships within the community, relationships among children and their informal interactions, conversations, and play. Data derived from participant observation helped in contextualizing children's behaviors, enriching the interpretation of interview data, and uncovering nuanced dynamics that shaped their knowledge about trees and the natural environment.

#### 2.2.3 Free Listing

To investigate children's knowledge of trees, we used the free listing method, a technique widely employed in the social sciences more than a decade (Purzycki 2011, 2016; Bernard and Ryan 2009; Hough and Ferraris 2010, Smith et al. 1995, Smith and Borgatti 1997, Oravecz et al. 2014; Romney et al. 1986). This method required 10 children from the combined class to list all the tree species they knew, regardless of their ability to identify them in a real‐world context. In collaboration with the school principal, we allocated several hours within the school premises for this research activity. Free listing is particularly beneficial because it captures participants' unprompted associations, offering a clear picture of their cognitive categories and cultural knowledge. It also reveals the relative salience of concepts, as more frequently mentioned species are likely the most familiar or significant to the children. Additionally, free listing allows flexibility in data collection, making it suitable for various age groups and contexts while minimizing the researcher's influence. This method provided insight into how children conceptualize trees in their environment and which species hold cultural or educational relevance. Free listing was also perceived as a fun and playful activity by the children. Data derived from free listing were instrumental in identifying the most culturally and experientially salient tree species for the children, highlighting patterns in their botanical knowledge and revealing gaps between formal education and everyday ecological understanding.

#### 2.2.4 Map Drawing

Each child was tasked with drawing a map of the trees that grow in the vicinity of their school. This method was crucial for understanding whether the most salient trees for this study group were those commonly found in their gardens. It was particularly engaging for the children, helping to build rapport and trust (Punch 2002). The children could focus solely on the playful activity of drawing, creating a more relaxed and open environment for communication.

#### 2.2.5 Individual and Group Walks

Walking outside the school building with the children was used to verify and deepen our understanding of their knowledge and recognition of tree species in situ. This research technique was particularly valuable for examining the relationship between children's knowledge of tree species names and their ability to recognize real trees in their environment. Additionally, the walks provided an opportunity for more general conversations about trees and plants, allowing for a more holistic view of how the children relate to and engage with the natural world.

#### 2.2.6 Semi‐Structured Interviews

Following the free listing method, we carried out semi‐structured interviews. The interview questions focused on the children's knowledge of trees, including where the trees grew, their appearance, characteristics, functions, and how the children learned about them. Additionally, the questions addressed the children's leisure activities, descriptions of their interactions with nature, family backgrounds, and relationships with family members. This data was essential in understanding how information about nature was believed to be transmitted from family or community members to the children. Each interview lasted approximately 20 min, during which we encouraged the children to initially speak freely about a topic of their liking. After such warm‐up, we proceeded with the interview questions.

#### 2.2.7 Diaries

During the study, the children were asked to write and describe how they spent their time after school. At the start, they received instructions on how to use their diaries and were expected to update them regularly, ideally each day throughout the research period. We acknowledge the limitations of this method (Punch 2002), as we did not verify the information recorded in the diaries and cannot confirm its accuracy. However, the diaries offered insight into how children spent their free time after school when the researcher could not be present. The primary goal was to determine whether the children spent time outdoors.

#### 2.2.8 Data Analysis

In analyzing ethnographic data—including notes from participant observations, interviews, and walks with children—we employed a qualitative, inductive approach (Thomas 2006), while acknowledging the challenges of justifying experience‐based conclusions (Bendassolli 2013). During fieldwork, VR kept a detailed field diary, serving as a ‘mnemonic device’ (Okely 2008). This article uses transcribed excerpts from these diaries to provide insights into the school classroom. While this approach risks anecdotalism (Silverman 2005), we mitigated concerns about representativeness by rigorously transcribing all handwritten notes and anonymizing participants. In the coding process, we used the grounded theory approach, supplemented by in vivo coding (Saldaña 2016). For the initial analysis, we employed a mixed method of open and in vivo coding. We carefully read through the data and highlighted sections that provided relevant information for our research topic. Then, we created paragraphs in the texts and derived codes from them. For example, when asked to describe the positive attributes of the school, we used open coding to create codes such as “smaller number of students,” “creative activities with children,” “opportunities to go to the forest,” and so on. For the question about informal education on tree species, recurring codes included the “need for parental involvement in education about tree species,” “extracurricular activities,” as well as in vivo codes that describe the work at the school more authentically. After creating the codes, we organized them into central categories using selective and axial coding, which represented the final version of the data analysis. The central categories identified in the interviews were: formal education, informal education, interpersonal relationships, positives of teaching, and negatives of teaching. To strengthen the validity and reliability of our findings, we implemented methodological triangulation by cross‐verifying insights from multiple qualitative sources: participant observation, interviews, free listing, diary entries, map drawings, and individual/group walks. This triangulation enabled us to compare patterns across different methods and contexts, reducing the risk of bias and enhancing the depth and credibility of our conclusions. For example, information about tree species learned at home or school was cross‐checked between interview narratives, observational notes, and children's map drawings or conversations during walks.

Data obtained from the free listing method were analyzed using R software for statistical computing. We used the AnthroTools package (Purzycki and Jamieson‐Lane 2017) to analyze the salience of different tree species. In this context, salience refers to the prominence or importance of the tree species as indicated by the order in which children listed them. Specifically, the salience score is based on the frequency with which each species was mentioned and the order in which it appeared in the free listing. The more frequently a species was listed or mentioned earlier in the sequence, the higher its salience score. In the literature, salience is commonly calculated using a method known as “Rank Frequency Analysis” (RFA), which combines both the frequency of mention and the position of the item in the list (Borgatti 1996). The salience score is often adjusted by considering both the order of mention (i.e., whether the tree species was mentioned early in the list) and the total number of participants who mentioned that species. This helps to provide a clearer indication of the relative importance or familiarity of each species within the group. For example, a species that is mentioned by many participants and early in their lists will have a higher salience score compared to species that are mentioned less frequently or later in the list. The higher the salience score, the more central the species is to the participants' mental representations of trees (see Supporting information S1).

This mixed‐method analytical strategy—integrating thematic and statistical analysis, inductive coding, and triangulation—allowed for a robust, multi‐dimensional understanding of how children perceive, learn about, and relate to trees in their everyday environments.

## 3 Results

### 3.1 Fruit Trees Dominate Children's Botanical Knowledge

The analysis of the free lists showed that the most salient species were the apple tree, cherry tree, and silver birch (see Table 1). Altogether, 10 children named 32 different tree species, 16 of which were fruit trees or shrubs commonly consumed at home, such as apples, pears, cherries, and peaches. The species mentioned first were typically those located near the school and thus easily identifiable by the children. Notably, a fourth‐grade girl listed the highest number of species, while the fewest were named by a second‐grade girl. On average, students in the combined second and fourth‐grade class named three species.

**TABLE 1:** Salience of different tree species.

	Tree species in Slovak	Tree species in English	Tree species in Latin	Salience score
1	Jabloň domáca	Apple Tree	*Malus domestica*	0.40
2	Čerešňa vtáčia	Cherry tree	*Cerasus avium*	0.30
3	Breza biela	Silver birch	*Betula pendula*	0.26
4	Hruška obyčajná	Pear tree	*Pyrus communis*	0.20
5	Buk lesný	European beech	*Fagus sylvatica*	0.15
6	Jedľa biela	Silver fir	*Abies alba*	0.15
7	Vŕba biela	White willow	*Salix alba* L	0.14
8	Černica (bot. ostružina černicová)	Blackberry	*Rubus fruticosus* agg	0.14
9	Dub letný	English oak	*Quercus robur*	0.14
10	Javor horský	Sycamore maple	*Acer pseudoplatanus*	0.14
11	Borovica lesná	Scots pine	*Pinus sylvestris*	0.13
12	Pagaštan konský	Horse chestnut	*Aesculus hippostanum*	0.13
13	Smrekovec opadavý	European larch	*Larix decidua*	0.12
14	Smrek obyčajný	Norway spruce	*Picea abies*	0.12
15	Ríbezle červené/biele…	Red/White currants	*Ribes rubrum*	0.12
16	Slivka domáca	Domestic plum	*Prunus domestica*	0.10
17	Lipa srdcovitá	Heart‐leaved linden	*Tilia cordata*	0.08
18	Brečtan obyčajný	English ivy	*Hedera communis*	0.07
19	Palma	Palm	*palma*	0.05
20	Marhuľa obyčajná	Apricot	*Armeniaca vulgaris*	0.03
21	Orech kráľovský	English walnut	*Juglans regia*	0.03
22	Olivovník (bot. oliva európska)	Olive tree	*Olea europaea*	0.02
23	Hrab obyčajný	Hornbeam	*Carpinus betulus*	0.01
24	Malina (bot. ostružina malinová)	Raspberry	*Rubus idaeus*	0.01
25	Citrovník pravý	Citron	*Citrus limon*	0.01
26	Vinič hroznorodý	Grapevine	*Vitis vinifera*	0.06
27	Broskyňa obyčajná	Peach	*Prunus persica*	0.027
28	Pomarančovník (bot. citrovník pomarančový)	Orange tree	*Citrus aurantium*	0.024615385
29	Jelša lepkavá	Common alder	*Alnus glutinosa*	0.017543860
30	Egreš (bot. ríbezľa egrešová)	Gooseberry	*Ribes uva‐crispa*	0.009230769
31	Čučoriedky (bot. brusnica čučoriedková)	Bleuberries	*Vaccinium myrtillus*	0.006153846
32	Tuja západná	Western red cedar	*Thuja occidentalis*	0.005847953

Age appeared to play a significant role in botanical knowledge. Although the sample size was small, a clear pattern emerged: second‐graders (aged 7) listed an average of 9 species, while fourth‐graders (aged 9) listed an average of 18. Among younger children, responses ranged from 1 to 16 species, whereas older children listed between 13 and 25. These differences likely reflect both cognitive development and the cumulative impact of direct experiences with nature over time. As older children had more years of formal education and informal outdoor engagement, they tended to recall and recognize a broader range of tree species.

Trees frequently cited by the children were those located around the school or in their households, while species outside their immediate vicinity were rarely mentioned. Species such as walnut, lemon tree, gooseberry, blueberry, and thuja were mentioned less frequently. The descriptions often reflected personal experiences; for example, children recounted how they collected and processed fruit from trees with their families, such as making jam. The horse chestnut (lat. *Aesculus hippocastanum* ) was mentioned in the context of making small toys, and the willow (lat. Salix) was associated with a specific location where the class once observed a “family of ducks.” Exotic trees were recognized based on personal experiences or memories, such as vacations by the sea or ornamental garden plants.

**FIGURE 2:**
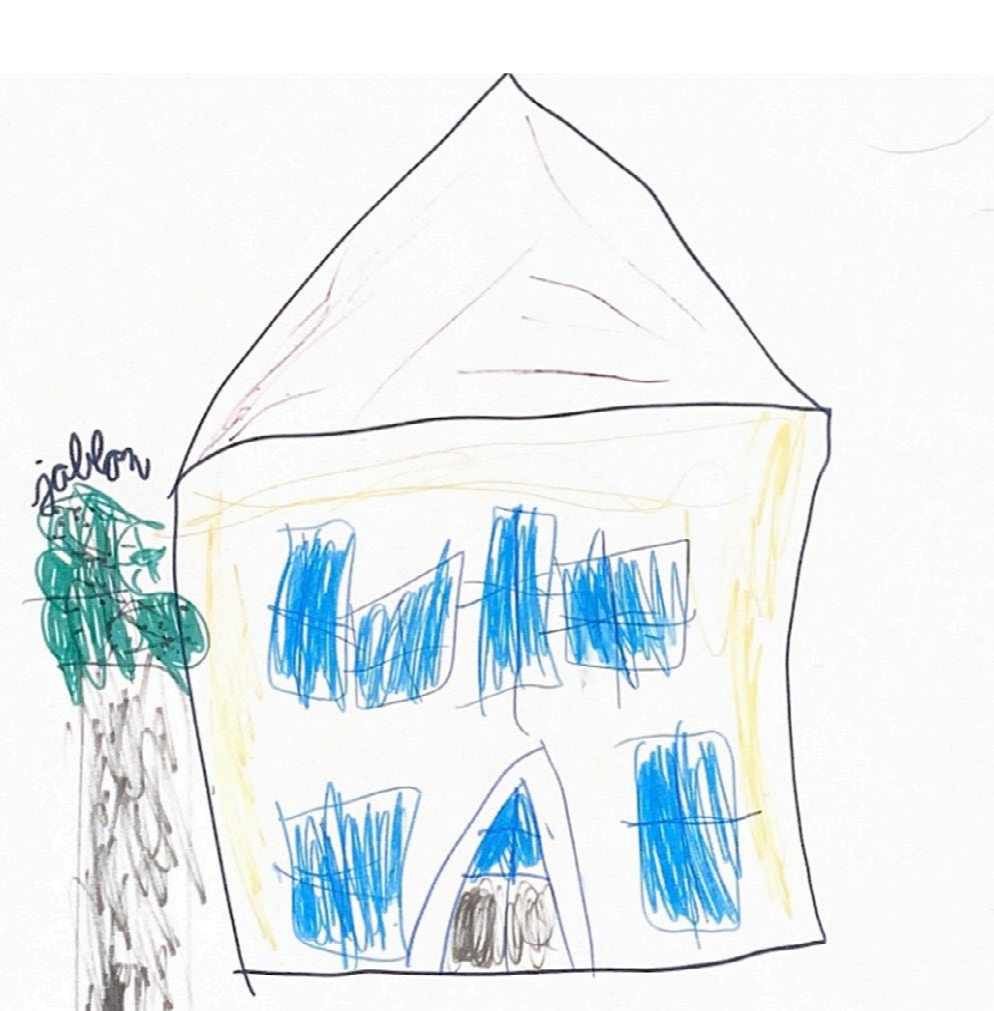
Nelka's illustration of the trees near the school. This is a copy of a picture drawn by Nelka (7 years old). It portrays the apple tree, the most salient of all tree species.

**FIGURE 3:**
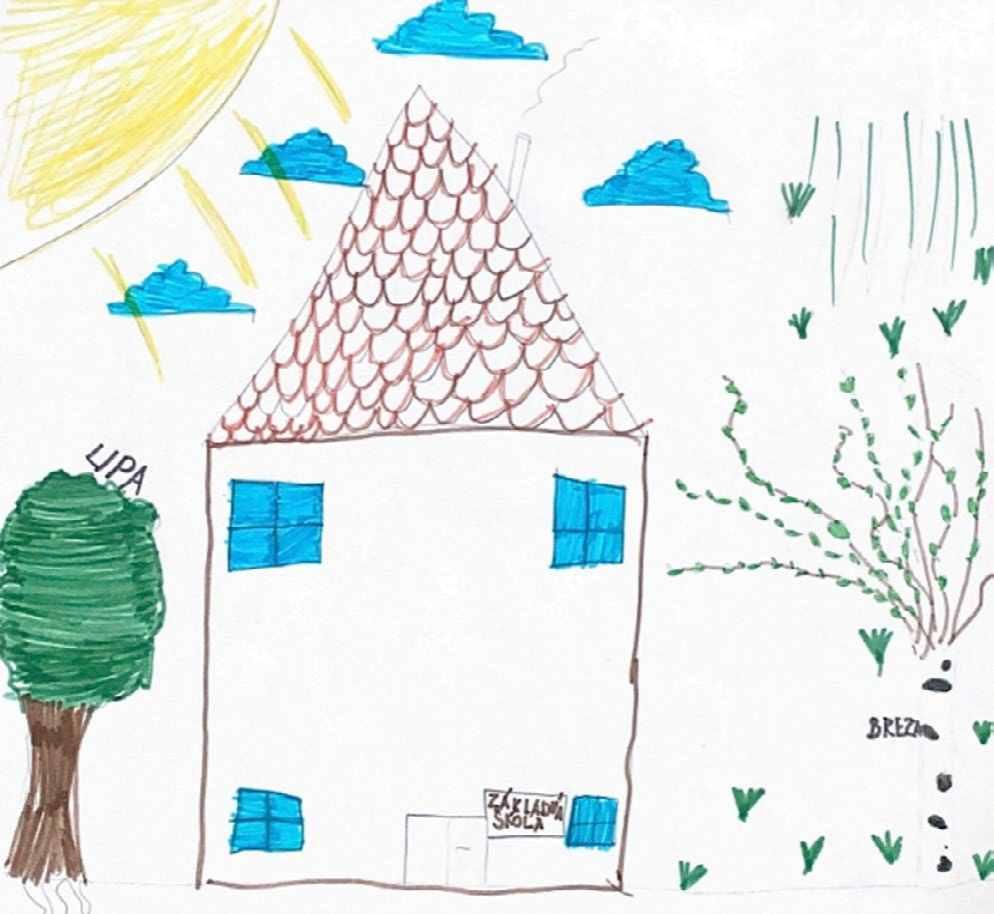
Salome's illustration of the trees near the school. Salome portrays a linden on the left and a birch on the right.

Salience of tree species was consistent with the maps drawing of the trees near the vicinity of the school. All the children focused on the most salient trees—the beech and the fruit trees, apple tree and a cherry tree (see Figures 2 and 3).

While fruit trees were the most frequently mentioned, the silver birch also held a notably prominent position in the children's responses. This may be attributed to its distinct and easily recognizable appearance—particularly its white bark and delicate leaves—which makes it visually memorable, even to young learners.

In contrast, the four tree species included in the formal school curriculum were not among the most salient in the free listing results. The European beech ranked fifth, the English oak ninth, the Scots pine eleventh, and the European hornbeam as low as twenty‐third. If formal instruction played a central role in shaping children's tree knowledge, these species would likely have appeared more frequently and earlier in their lists. Instead, species more closely tied to children's lived experiences—especially fruit‐bearing trees encountered at home or during shared family activities—were mentioned more often and more prominently. This suggests that experiential familiarity exerts a stronger influence on botanical knowledge than curriculum exposure alone.

Despite their ability to identify and name familiar and visually distinctive trees, most children demonstrated limited understanding of ecological processes, particularly those related to tree reproduction. Their knowledge appeared to be grounded more in practical, everyday interactions than in scientific or biological concepts. These gaps in ecological understanding are explored in greater detail in the following section.

### 3.2 Limited Understanding of Ecological Processes

Children were able to distinguish between coniferous and deciduous trees and recognized that trees have a trunk and crown, setting them apart from other plants. However, their understanding of tree reproduction was limited. Some children knew that planting a seed is necessary but lacked comprehension of how the process works, while others had no knowledge of it at all. When asked about the functions of trees, the most common response highlighted their role in producing oxygen. Shrubs were generally considered less important, mainly due to their smaller size. Children believed that because shrubs are smaller, they produce significantly less oxygen than trees, making trees seem far more essential. The second function of trees mentioned was their role as a source of wood, used for making furniture, objects, and providing shelter for animals. The function of shrubs, on the other hand, was rarely mentioned.

Children could recognize fruits, flowers, leaves, and needles, matching them with the correct names of tree species. They understood the basic structure of trees and could name all the parts. Some fourth‐grade children demonstrated deeper knowledge of specific coniferous trees, describing the shape and length of needles, the size and shape of cones, and the typical environments where they grow. This more extensive knowledge was likely due to formal education, as the research was conducted at the end of the school year after they had completed the first stage of elementary school.

Children also displayed knowledge about the essentials for tree survival. “Trees need soil, water, sun, and air to survive,” said Simonka (9 years old). “Water, nutrients, soil, and light are important,” added Lilianka (9 years old).

Children described the functions of trees more extensively than those of shrubs, with every child mentioning oxygen production as the first function. “We use trees for air, as fuel, or wood is used to make furniture,” said Salome (9 years old). “We use trees for oxygen, but I don't know what shrubs are good for,” remarked Lilianka (9 years old). “Trees produce oxygen and are homes for animals. The function of shrubs is food for both humans and animals, and their wood is used for heating. Trees are used to make furniture, paper, and pencils,” explained Simonka (9 years old).

There were noticeable differences based on grade level. Second‐grade children often used abbreviated names for trees and occasionally used a local dialect. For example, they referred to currants as “viničky” instead of the standard Slovak term “ríbezle.” In contrast, fourth‐grade children more frequently used full, standard names for trees, showing the impact of formal education.

All second‐grade children expressed a desire to protect trees and forests. They understood that leaves, branches, and other parts of trees should not be removed unnecessarily. For example, one child said, “Trees need to be protected. Don't tear leaves off them unnecessarily because it hurts the tree” (Sárka, 7 years old), while another remarked, “We need to protect trees, not tear them because they produce oxygen for us” (Edko, 7 years old). However, this protective attitude was not always reflected in their actions, as children sometimes ignored these guidelines and used tree parts in their play.

When asked, “How do trees reproduce?” their answers varied:

Trees are made by bees. (Nelka, 7 years old)

We plant a seed and a tree grows. (Edko, 7 years old)

I don't know. We plant it and it grows by itself. (Filip, 7 years old)

I don't know. I haven't learned that. (Ninka, 7 years old)

In contrast, when asked to explain what trees need to survive, their answers were more precise:

They need water, air, and care. (Barborka, 7 years old)

They need air, water, and soil. (Edko, 7 years old)

They need water. (Ninka, 7 years old)

They need water, air, nutrients, and clay. (Sárka, 7 years old)

Although children demonstrated a basic understanding of ecological concepts and a strong awareness of the importance of trees, their limited knowledge of reproduction suggests that experiential and contextual learning may be lacking. The following section explores how the quality of children's engagement with plants, rather than just time spent outdoors, influences their depth of botanical knowledge.

### 3.3 Quality of Outdoor Engagement Matters More Than Quantity

Spending time outdoors is valuable, but active engagement with plants significantly enhances children's knowledge of them. In total, the children documented 315 h of leisure time, with each child spending an average of 1 h and 24 min outdoors daily, equating to 9 h and 48 min per week. Table 2 captures each child's total recorded hours, average weekly time spent outdoors, and average weekly time spent on other activities.

**TABLE 2:** Reported time allocation of children's time spent outdoor.

Child	Age	Total recorded hours	Average weekly time spent outdoors	Average weekly time spent on other activities
Filip	7	64 h, 30 min	26 h, 33 min	141 h, 27 min
Salome	9	43 h	15 h, 50 min	152 h, 10 min
Edko	7	40 h	16 h, 28 min	151 h, 32 min
Ninka	7	39 h	14 h, 22 min	153 h, 38 min
Simonka	9	30 h	10 h, 30 min	157 h, 30 min
Lilianka	9	30 h	12 h, 21 min	155 h, 39 min
Nelka	7	27 h	9 h, 57 min	158 h, 3 min
Sárka	7	26 h, 30 min	11 h, 36 min	156 h, 24 min
Barborka	7	15 h	8 h, 45 min	159 h, 15 min

*Note:* This table captures each child's total recorded hours, average weekly time spent outdoors, and average weekly time spent on other activities.

Filip and Salome reported spending the most time outdoors, yet their knowledge of trees was among the weakest. Similarly, Nelka, Sárka, and Barborka—who spent the least time outside—also demonstrated limited understanding of tree species. In contrast, Edko, Ninka, Simonka, and Lilianka, who not only spent time outdoors but also engaged in nature‐related activities with their parents or grandparents, showed a notably deeper understanding of trees.

These children participated in hands‐on experiences such as gardening, fruit harvesting, and nature walks, which significantly enriched their botanical knowledge. For example, Simonka (9 years old) described a rich array of nature‐related activities:

I go to the forest with my parents, we go mushroom picking, we go for walks to the swing near Granč (a natural swing between two trees), and with my dad we also go to the hunting stand to observe nature… We visit my grandma, I help her take care of the garden, I really enjoy baking with her—last time we made cherry compote.

Likewise, Lilianka (9 years old) emphasized the importance of her time spent with her grandmother:

I go for walks with my grandma, we gather various herbs there, for example for tea, I help her in the garden, we bake cakes together, and we take care of the little garden.

These accounts illustrate that what matters most for developing botanical knowledge is not simply how much time children spend outdoors, but how they spend that time—and with whom. Children who engage in culturally meaningful, purposeful, and relational activities with plants demonstrate a stronger and more nuanced understanding of tree species.

Simply spending time in nature, without meaningful interaction, did not significantly impact children's plant knowledge. Those who demonstrated the most comprehensive understanding were actively involved in plant‐related tasks such as watering gardens, planting trees and shrubs, and collecting herbs. Verbal explanations from family members and the use of educational resources like plant encyclopedias further supported their learning.

In contrast, children who spent time outdoors without engaging in educational activities—such as cycling with parents without discussing plants—showed less understanding of tree care. Similarly, those present in gardens but not actively involved in gardening tasks also exhibited limited botanical knowledge.

Beyond hands‐on interaction with plants, peer interaction also played an important role in shaping knowledge. The following section explores how children shared and reinforced botanical concepts through informal conversations and social learning.

### 3.4 Peer Learning and Informal Knowledge Sharing

The analysis of participant observation in our study revealed that after biology class, during breaks, children often discussed the topics covered in class or explained concepts that were not yet part of the curriculum to younger students. This type of interaction, particularly with the structured age groups of 7 and 9 years old, may have accelerated the specific knowledge transmission. Children also talked about plants and exchanged knowledge outside of the formal curriculum.

A vivid example comes from an observation in the school club:

In the school club, the children worked on their homework together. Since the second‐ and fourth‐graders spend the most time together in the classroom, they naturally sat close to one another while writing and often talked among themselves.

Nelka (a second‐grader) was working on a revision worksheet (as the school year was nearing its end), but she couldn't tell the difference between a spruce and a fir tree. Her classmate Salome (a fourth‐grader) offered to show her what a fir and a spruce look like in the schoolyard after they finished their homework. After completing their assignments, the children—as usual—moved to the playground in front of the school, where they played until their parents came to pick them up. Later, Salome took Nelka over to the trees and showed her the fir tree. She explained that fir trees don't prick and have long needles, which makes them easy to remember and distinguish from other trees. (Fieldnotes, VR, 16.6.2023).

This instance illustrates how informal peer instruction was embedded in the children's daily routines and social relationships. It also highlights that such interactions were not limited to academic content but extended into the natural environment around the school.

For children, peer opinions often carry more weight than those of adults, such as parents or teachers (Hirschfeld 2002). This could be linked to the fact that, for most of our evolutionary past, children grew up in mixed‐age and mixed‐sex groups. Research from an evolutionary anthropology perspective highlights the significance of peer‐to‐peer learning in transmitting essential survival skills, including plant identification, tool‐making, and social norms. Studies have shown that children in such environments are highly attuned to their peers, with much of their learning occurring through observation, imitation, and hands‐on practice (Lancy 2012; Lew‐Levy et al. 2017).

## 4 Discussion

1. One of the key findings of this study is that children are more likely to remember tree species when their experiences with those trees are connected to personal memories shared with family or friends, or tree species they directly use or consume. This suggests that it is not just the amount of time spent outdoors, but the quality of those interactions that significantly influences plant knowledge. These results align with previous studies on plant awareness. For instance, Stagg and Dillon (2022) emphasized that people's plant awareness develops through frequent interactions with plants that are directly relevant to their lives (see also Guerra et al. 2024; Linderwell et al. 2024; ). Similarly, Lohr and Pearson‐Mims (2005) highlight the role of childhood memories of active plant interactions in fostering plant awareness. Moreover, Villarreal et al. (2018) argue that proximity to and engagement with plant life are critical to people's understanding of plants.
2. This study has also provided a glimpse into the role of peer‐to‐peer learning in the transmission of knowledge about trees. While informal learning is typically seen in non‐formal settings, children in formal school environments also engage in peer interactions that provide valuable opportunities for teaching and learning from each other. These exchanges play a vital role in enhancing learning beyond the structured curriculum. Therefore, it is essential to explore peer‐to‐peer teaching and knowledge transmission within and outside classrooms, particularly around topics like plants and the natural environment, which often emerge informally after class when these subjects have been addressed. Further research into plant knowledge learning in peer groups within the informal settings of formal schools is needed to better understand its significance. Furthermore, incorporating this perspective into educational curricula could enhance children's plant knowledge acquisition. Schools should plan for and incorporate spaces that foster informal learning opportunities for peer groups, enriching the learning experience and promoting deeper engagement with the natural world.
3. A recent systematic review of place‐based education by Yemini et al. (2023) highlights the pedagogical value of grounding learning in the local biophysical, sociocultural, psychological, and political‐economic dimensions of place. While much of this literature is based in Anglophone or urban‐centered contexts, fewer studies explore rural environments or post‐socialist regions in Central and Eastern Europe. This study offers a unique contribution by situating its research in a small village in eastern Slovakia—a region where close relationships with nature, family‐based subsistence gardening, and informal ecological knowledge are still common. By focusing on children's interactions with trees in this rural context, the research not only addresses the biophysical and sociocultural dimensions of place‐based learning but also offers insights into how such environments foster ecological literacy outside of traditional, urban‐centered schooling models.

### 4.1 Limitations

While this study offers valuable insights into children's knowledge of trees and the educational and social factors shaping it, several limitations should be acknowledged. First, the research was conducted during late spring and early summer—a period when many trees are in full bloom or fruiting. This seasonal context likely influenced children's recall and recognition of certain species, particularly fruit trees that were visually prominent or actively used at the time. Consequently, salience scores and tree identification may reflect short‐term, seasonal familiarity rather than deeper or long‐term botanical knowledge. Second, some responses—especially those related to tree protection or pro‐environmental attitudes—may have been influenced by social desirability bias, with children providing answers they believed would please adults (e.g., “we shouldn't tear leaves because it hurts the tree”). Although participant observation helped contextualize and interpret these responses, there remains a possibility that children's verbal statements overstated their actual behaviors or beliefs. Third, the study relied in part on self‐reported data from children's diaries to assess how they spent time outdoors. While these diaries offered valuable insight into leisure activities, the information was not independently verified and may be subject to recall bias or selective reporting, particularly for routine or less memorable experiences. Finally, while the small sample size allowed for in‐depth ethnographic engagement, it limits the generalizability of the findings beyond this specific school and community context. Future research would benefit from comparative studies involving larger, more diverse populations, as well as investigations across different seasons, age groups, and geographic settings to better account for ecological variation and broader cultural influences.

### 4.2 Conclusion

This study offers a rare ethnographic perspective on plant knowledge among primary school children in a rural Eastern European context—a region and age group underrepresented in current literature. Unlike many studies that focus solely on classroom interventions, our approach integrated methods from ethnobotany, anthropology, and environmental education. We highlight the importance of informal learning pathways, including peer learning, cultural knowledge, and family practices, and demonstrate that rural environments are not automatically synonymous with botanical literacy.

The results can be summarized as follows:

- Fruit trees dominate children's botanical knowledge.
- Proximity and personal experience shape tree recognition.
- Quality of outdoor engagement matters more than quantity.
- Age influences the number of tree species children can name.
- Children have limited understanding of ecological processes.
- Peer interaction supports informal botanical learning.

The granularity of our data—including diary entries, child‐drawn maps, and free‐listing—reveals that even within a single classroom, children's knowledge is highly varied and deeply dependent on their socio‐familial context. These findings contribute depth to current understandings of plant awareness disparity and underscore the need for culturally and locally sensitive approaches in environmental education.

One of the clearest patterns—reflected in both the free listing and map drawing exercises—is the prominence of fruit trees such as apple and cherry. These species were not only physically present in children's environments but also featured in meaningful family routines, such as harvesting and cooking. This supports the conclusion that personal, hands‐on engagement increases species recognition and recall, reinforcing earlier claims in plant awareness research (Guerra et al. 2024; Linderwell et al. 2024; Jose et al. 2019; Stagg and Dillon 2022; Stagg et al. 2024; Lohr and Pearson‐Mims 2005).

Additionally, children who engaged in gardening, nature walks, or plant‐related activities with parents or grandparents demonstrated stronger tree knowledge than peers who spent similar amounts of time outdoors without such interaction. This suggests that the quality of engagement with nature outweighs the quantity of exposure, a point well supported in the ecological literacy literature (Orr 1992). In this context, incorporating direct engagement with plants and the natural environment into the school curriculum is essential for increasing children's appreciation of plants. Such interactions not only enhance plant knowledge but also boost creativity, independence, and self‐confidence in children (Boileau and Dabaja 2020); improve their health and well‐being (Malone and McLachlan 2023); support imaginative play (Dowdell et al. 2011); and improve academic achievement (Harvey et al. 2020).

Peer‐to‐peer learning also emerged as a critical but often overlooked dimension of informal education. Observations showed that older children frequently shared plant knowledge with younger peers during breaks or outdoor activities—illustrating a sociocultural learning process aligned with evolutionary perspectives on knowledge transmission (Hirschfeld 2002; Lew‐Levy et al. 2017). This supports recent calls to explore how informal, horizontal learning dynamics within schools can enrich formal curricula (Yemini et al. 2023).

These findings reinforce the value of place‐based education that centers on local ecologies and community practices (Sobel 2004; Gruenewald 2003). In a setting like Granč‐Petrovce—where children are embedded in agrarian and ecological rhythms—school‐based learning can be significantly enriched by drawing on the surrounding cultural and natural resources. To reduce plant awareness disparity, educators should incorporate outdoor, multisensory experiences that connect personal memory, physical interaction, and scientific understanding.

In sum, by tying children's cognitive development to direct experience, peer influence, and local environments, this study offers practical and theoretical contributions to the fields of environmental education, ethnobiology, and childhood learning. Future research could expand on this work by exploring seasonal variation in plant knowledge, conducting longitudinal studies across school years, or comparing urban and rural learning contexts.

Ultimately, this study reinforces the argument that plant awareness cannot be fostered through formal education alone. Instead, children's botanical literacy emerges through the intersections of school curriculum, family practice, peer interaction, and the everyday landscape. Outdoor learning must go beyond exposure to involve active, guided, and contextually meaningful engagement. Bridging formal education with cultural and familial practices—including opportunities for peer‐led learning and memory‐making with plants—may reduce plant awareness disparity and foster long‐term ecological stewardship.

## Ethics Statement

The research was approved by the Research Approval Committee of the Institute of Social Anthropology, Faculty of Social and Economic Sciences, Comenius University in Bratislava in May 2023. To all parents or legal guardians, informed consent forms were distributed with information on research goals and aims. Participants could withdraw from the research at any time. All personal data were fully anonymized; names of the children are pseudonyms. Interviews with children were not recorded. All interviewed participants were properly informed about the research topic, subjects, and goals.

## Conflicts of Interest

The authors declare no conflicts of interest.

## Supporting information


**Data S1:** pei370090‐sup‐0001‐dataset.xlsx.

## Data Availability

Data will be available online.
